# Performance analysis of hybrid deep learning framework using a vision transformer and convolutional neural network for handwritten digit recognition

**DOI:** 10.1016/j.mex.2024.102554

**Published:** 2024-01-05

**Authors:** Vanita Agrawal, Jayant Jagtap, Shruti Patil, Ketan Kotecha

**Affiliations:** a Department of Computer Science and Information Technology, Symbiosis Institute of Technology, Symbiosis International (Deemed University), Pune, Maharashtra, India; b NIMS Institute of Computing, Artificial Intelligence and Machine Learning, NIMS University Rajasthan, Jaipur, India; c Symbiosis Centre for Applied Artificial Intelligence (SCAAI), Symbiosis Institute of Technology, Symbiosis International (Deemed University), Pune, Maharashtra, India; d UCSI University, Kuala Lumpur 56000, Malaysia

**Keywords:** Convolutional Neural Network, Vision Transformer, Handwritten Digit Recognition, Machine Learning, Computer Vision, Convolutional vision transformer

## Abstract

Digitization created a demand for highly efficient handwritten document recognition systems. A handwritten document consists of digits, text, symbols, diagrams, etc. Digits are an essential element of handwritten documents. Accurate recognition of handwritten digits is vital for effective communication and data analysis. Various researchers have attempted to address this issue with modern convolutional neural network (CNN) techniques. Even after training, CNN filter weights remain unchanged despite the high identification accuracy. As a result, the process cannot flexibly adapt to input changes. Hence computer vision researchers have recently become interested in Vision Transformers (ViTs) and Multilayer Perceptrons (MLPs). The shortcomings of CNNs gave rise to a hybrid model revolution that combines the best elements of the two fields. This paper analyzes how the hybrid convolutional ViT model affects the ability to recognize handwritten digits. Also, the real-time data contains noise, distortions, and varying writing styles. Hence, cleaned and uncleaned handwritten digit images are used for evaluation in this paper. The accuracy of the proposed method is compared with the state-of-the-art techniques, and the result shows that the proposed model achieves the highest recognition accuracy. Also, the probable solutions for recognizing other aspects of handwritten documents are discussed in this paper.

• Analyzed the effect of convolutional vision transformer on cleaned and real-time handwritten digit images.
• The model's performance improved with the implication of cross-validation and hyper-parameter tuning.
• The results show that the proposed model is robust, feasible, and effective on cleaned and uncleaned handwritten digits.

## Graphical abstract

**Figure:**
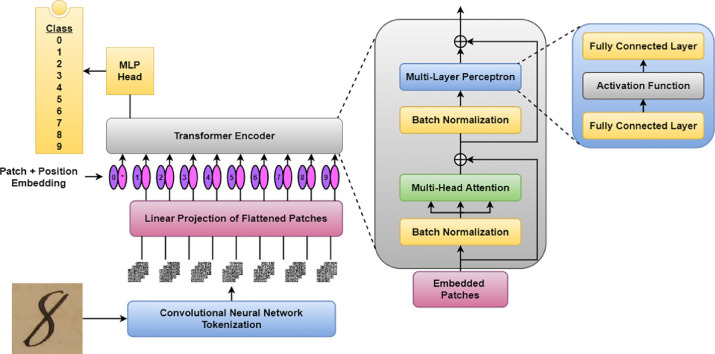

Specifications table

**Table:** 

Subject area:	Engineering
More specific subject area:	Computer Vision
Name of your method:	Convolutional vision transformer
Name and reference of original method:	H. Wu, B. Xiao, N. Codella, M. Liu, X. Dai, L. Yuan, L. Zhang, Cvt: Introducing convolutions to vision transformers, in: 2021 IEEE/CVF International Conference on Computer Vision (ICCV), 2021, pp. 22–31. doi:10.1109/ICCV48922.2021.00009.
Resource availability:	Data: https://didadataset.github.io/DIDA/; https://pytorch.org/vision/main/generated/torchvision.datasets.EMNIST.html Software: Google Colab, PyTorch library

## Method details

**Fig. 1:**
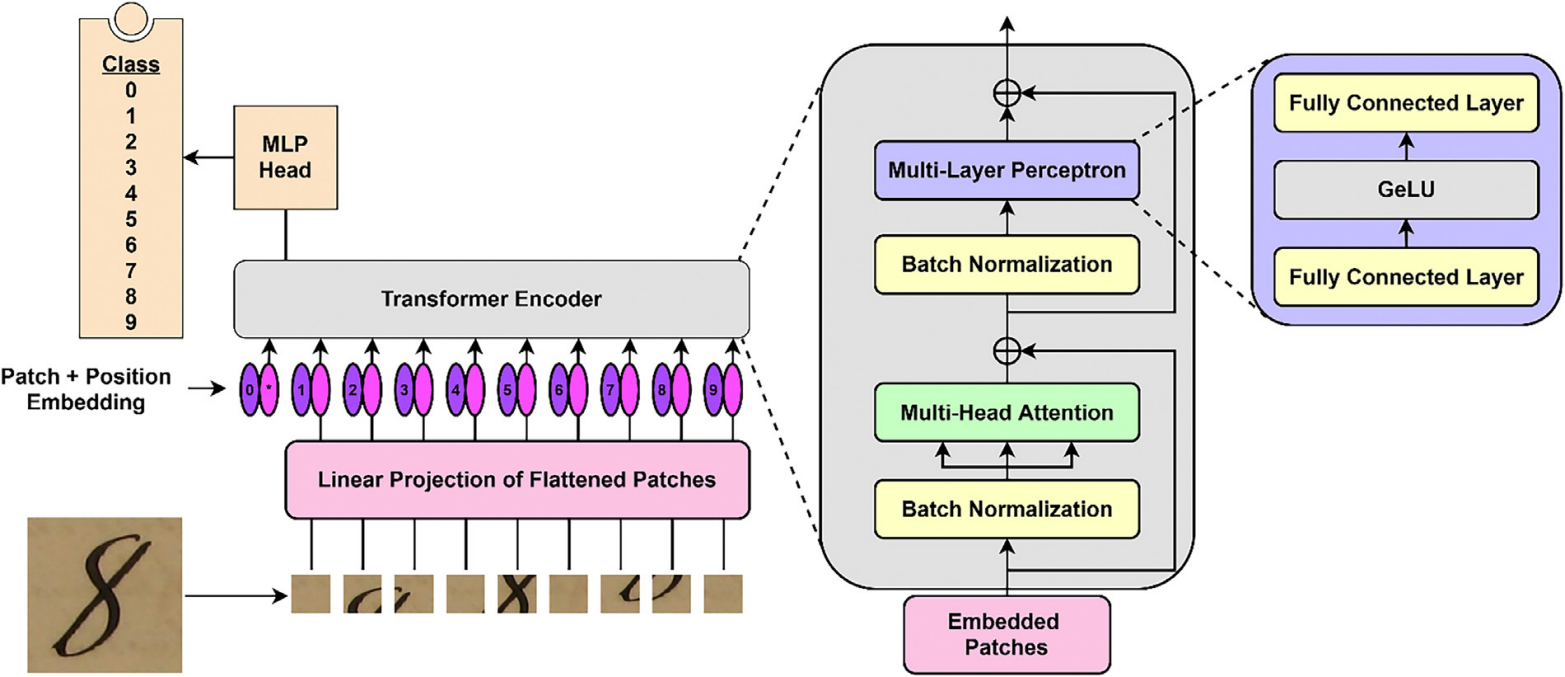
The overview of ViT Architecture.

The construction of the vision transformer is depicted in the Fig. 1 [1]. The transformer receives embedded patches as input. The embeddings are constructed by dividing the image into patches of the same size. Padding accounts for the disparity between patch size and image size. Since the transformer requires a 1D sequence as input, the patches are projected onto a 1D vector. The position of patches is learned through positional embedding. Each layer is subjected to layer normalization [2]. Eq. (1) provides the formulas for layer normalization for 1D sequence vector ϑ.

(1) $$\left(\vartheta \right)=\gamma \frac{\vartheta -\mu }{\sigma }+\beta$$

where,

The average of the elements in v is μ.
The standard deviation of the components in v is σ.
The scaling parameter is γ, and
A biased vector parameter is β.

Self-attention is the mechanism employed in multi-head attention. A query (*Q*), key (*K*), and value are provided as the input to self-attention (*V*). To the output, it maps *Q* and *K*-*V* pairs. The softmax function calculates the weight of the value vector. The definition of attention is given in Eq. (2) [3].

(2) $$Attention\left(Q,K,V\right)=softmax\left(\frac{QK^{T}}{\sqrt{d}}\right)V$$

where *d* stands for the secret dimensions.

The MLP-head layer divides the data into smaller chunks and calculates the attention of each head independently and simultaneously. Gaussian error linear unit (GeLU) activation function is used in the two-layer feed-forward MLP layer as shown in Eq. (3) [4].

(3) $$GeLU\left(x\right)=x\varphi \left(x\right)\approx 0.5x\left(1+\tanh\left[\sqrt{2/\pi }\left(x+0.044715x^{3}\right)\right]\right)$$

where the Gaussian cumulative distribution is denoted by φ(x).

The largest historical handwritten digit dataset (DIDA) [5,6] has 250,000 images, and the Extended Modified National Institute of Standards and Technology (EMNIST) digits dataset [7] comprises 280,000 images. This study employs the hybrid convolutional vision transformer (CViT) [8] to recognize handwritten numbers.

The images are first scaled to ensure that every image in the dataset is the same size before it is delivered to the model. The effectiveness of the model is improved with it. The photos are then standardized with the help of mean and standard deviation. Rescaling is another normalization name, making using the same procedure on all images easier. All photos are scaled, which results in a constant learning rate.

Wu et al. [8] proposed the CViT architecture. Three stages make up the architecture. We changed the transformer's parameters to accommodate handwritten digit datasets. To increase model performance and accuracy, the value of the depth parameter is modified in particular. The generation of embedding patches and feature extraction are the two primary uses of CNN.

## Method validation

### Datasets

**Fig. 2:**
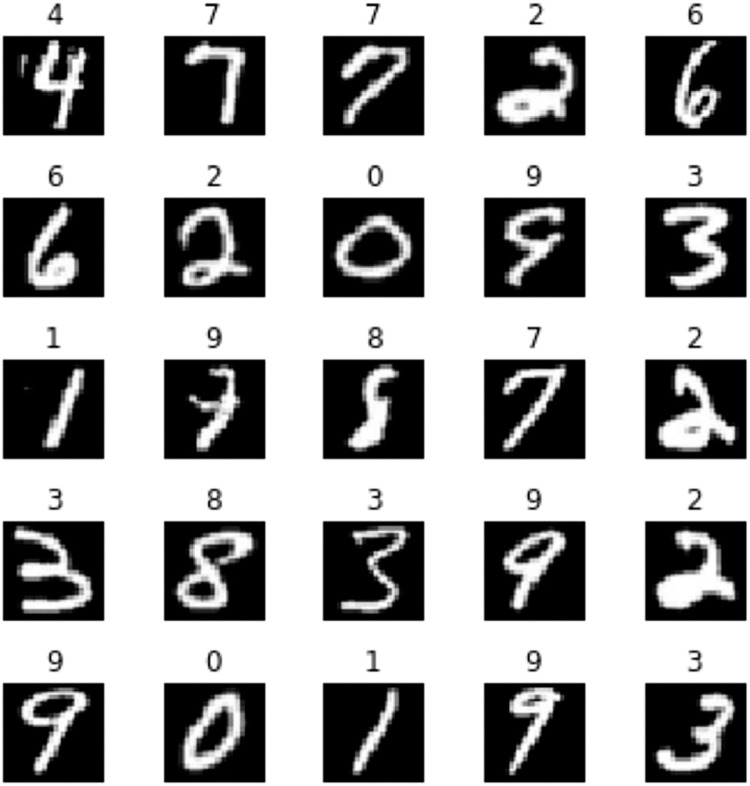
Digit samples from the EMNIST dataset.

The EMNIST-digit and DIDA benchmark datasets are used for experimentation. There are 280,000 images in the EMNIST-digit dataset, of which 252,000 are used for training and 28,000 for testing. Images used for training and testing are altered after each fold as cross-validation is used. The data set is split into ten balanced classes, numbered 0, 1, 2, 3, 4, 5, 6, 7, 8, and 9. Fig. 2 shows a sample image of this dataset.

**Fig. 3:**
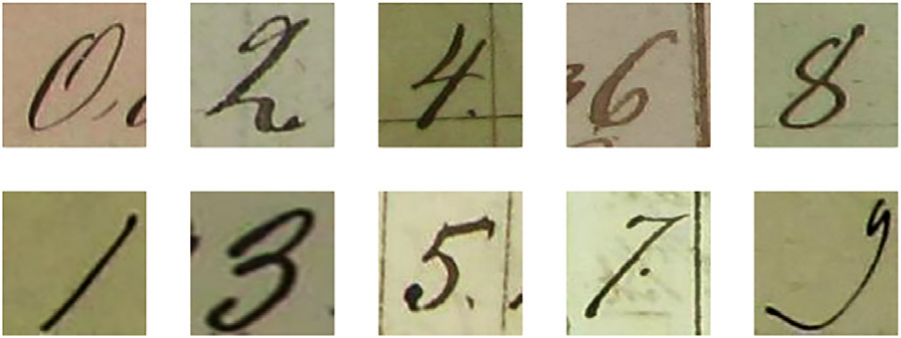
Samples of digits in the DIDA dataset.

EMNIST-digit database images have been size-normalized, denoised, and cleaned. DIDA, a different benchmark dataset, is therefore employed to evaluate the suggested model. The images of DIDA are uncleaned. Two hundred fifty-two thousand eight hundred sixty photos cropped from historical documents make up the DIDA dataset. The writing styles, sizes, orientations, widths, and layouts of the digits in the DIDA dataset vary. There are ten classes in the DIDA dataset as well. Fig. 3 displays an example image of each category.

### Model parameters and performance metrics

Choosing the optimal hyper-parameter value for the framework is an especially crucial step. Optuna [9] is a hyper-parameter optimizing methodology for black-box optimization methods and machine learning. As a black-box optimizer, Optuna evaluates the behavior of the hyper-parameters. The optuna trial object specifies the type and scope of the hyper-parameters that must be tweaked. The [“Adam,” “RMSprop,” or “SGD” optimizer is selected. The backpropagation learning values range from 0.00001 to 0.1 in a logarithmic fashion. With a scaling factor of 16, the batch size is between 16 and 256.

The optimizer, stochastic gradient descent (SGD), with a learning rate of 0.01 and batch size of 192, was suggested by the Optuna application on the EMNIST dataset. The ideal dropout value found was 0.25. The PyTorch library is used for experimentation and classification with the logarithmic softmax function. The negative log-likelihood (NLL) loss is used to calculate the loss.

### Results and discussion

**Fig. 4:**
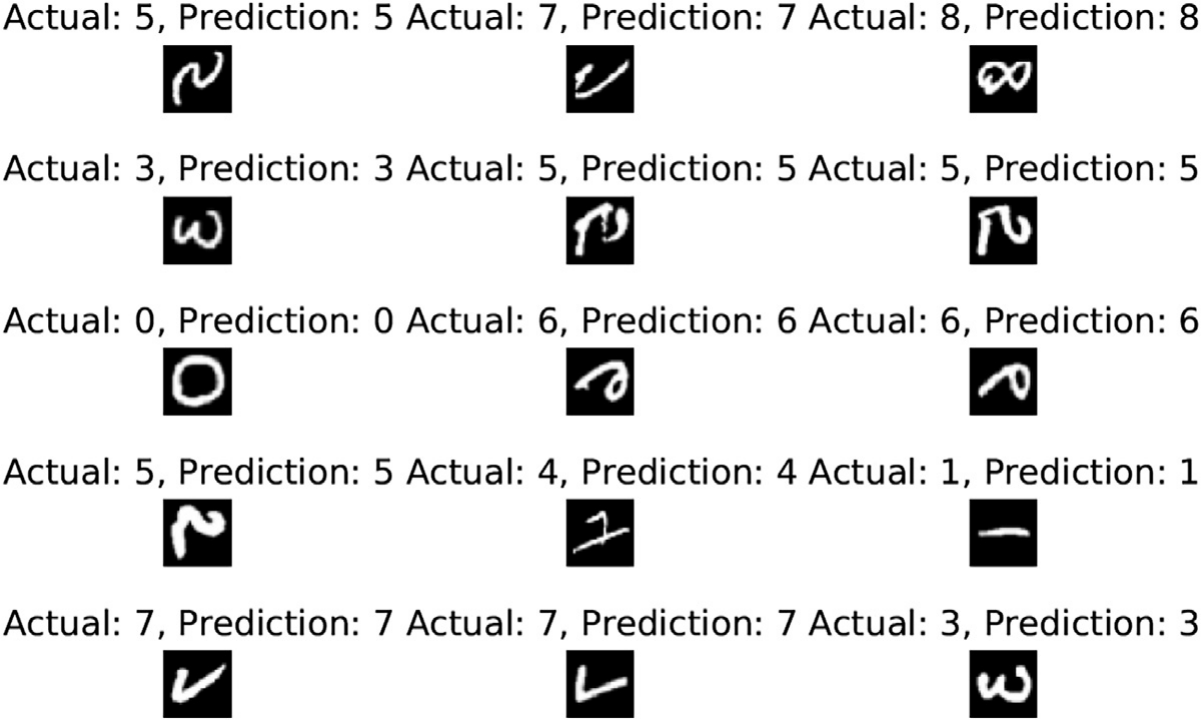
Recognition result of handwritten digits from EMNIST-digit dataset.

Due to the sparse data, the experiment uses K-fold cross-validation with ten folds to prevent overfitting. On the EMNIST-digit dataset, the overall 10-fold accuracy is 99.89 %. The accuracy, precision, recall, and f1-score are all 99.89 %. A maximum accuracy of 99.95 % was attained in the most recent fold. The model's ROC AUC score is 99.99 percent. Fig. 4 demonstrates the results for the EMNIST dataset, where a few examples with their actual, predicted, and image values are displayed.

**Fig. 5:**
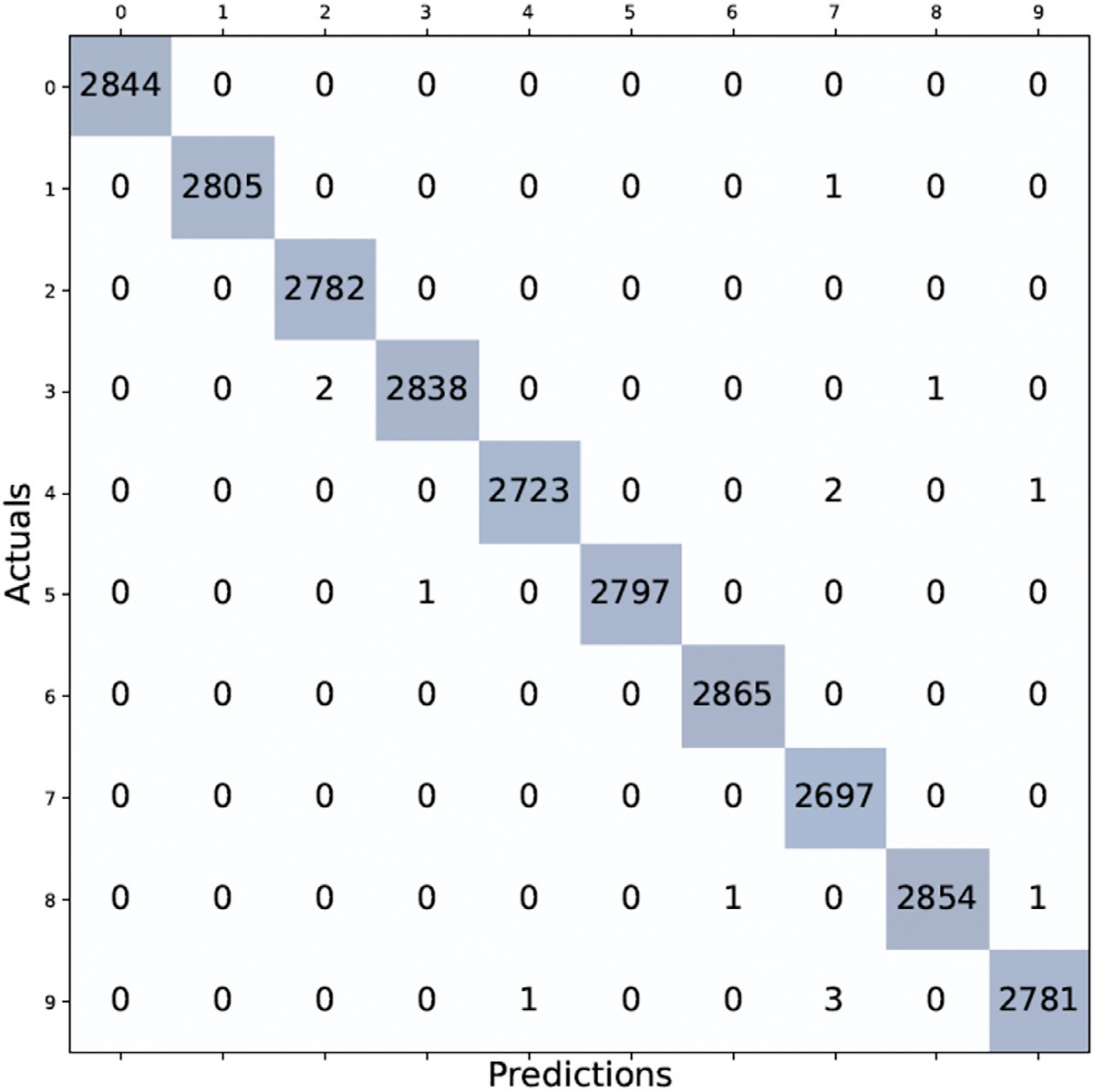
Confusion matrix results on EMNIST-digit dataset.

Fig. 4 demonstrates how the framework can accurately predict the outcomes of rotated images. The final fold's confusion matrix testing results are depicted in Fig. 5. The count of accurate predictions broken down by class is shown in the blue box.

**Fig. 6:**
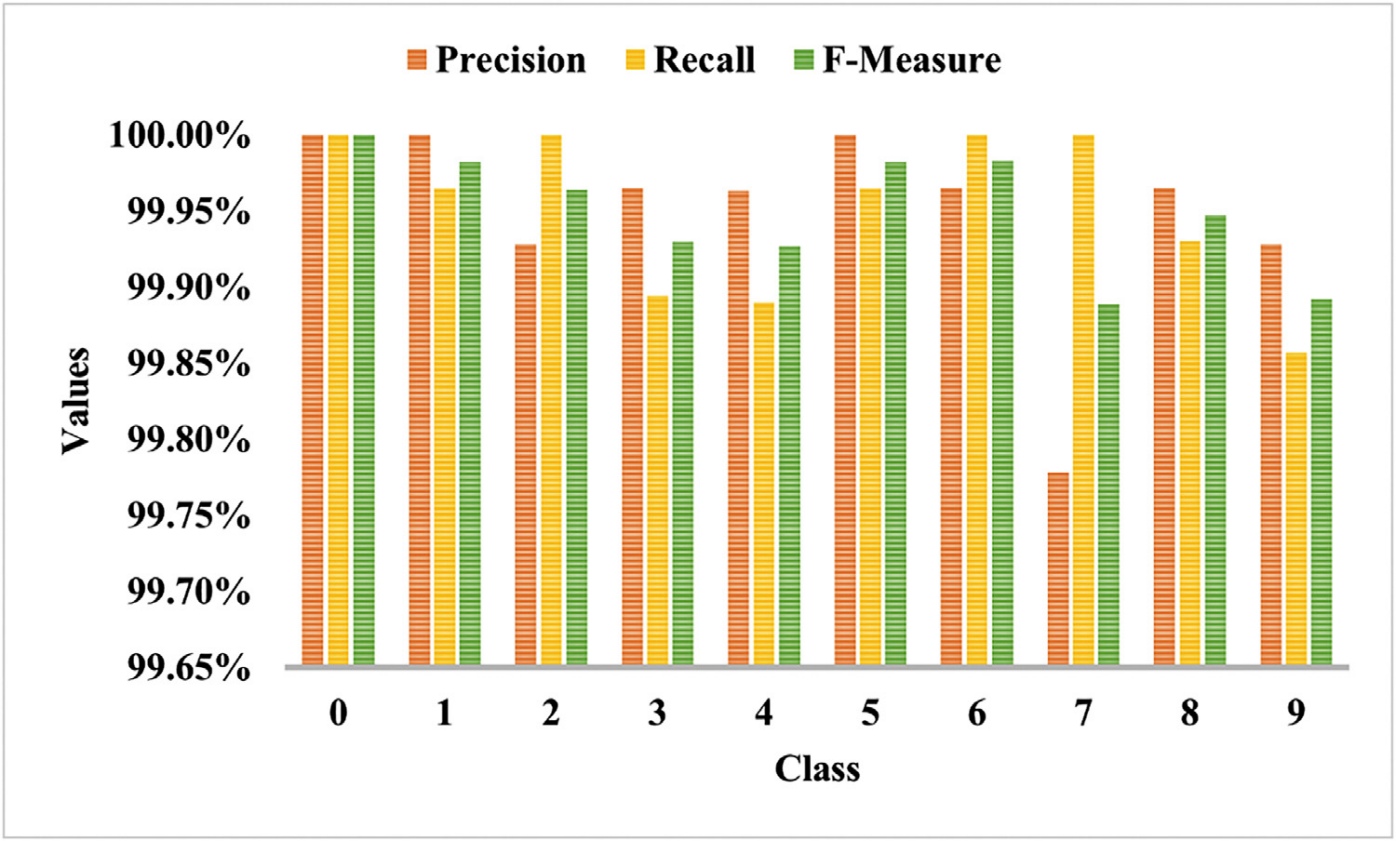
Class-wise comparison of performance metrics on the EMNIST dataset.

Fig. 6 shows the classwise precision, recall, and f-measure analysis. The proposed model can classify digits 0, 1, 2, 5, 6, and 8 more accurately than 3, 4, 7, and 9.

**Fig. 7:**
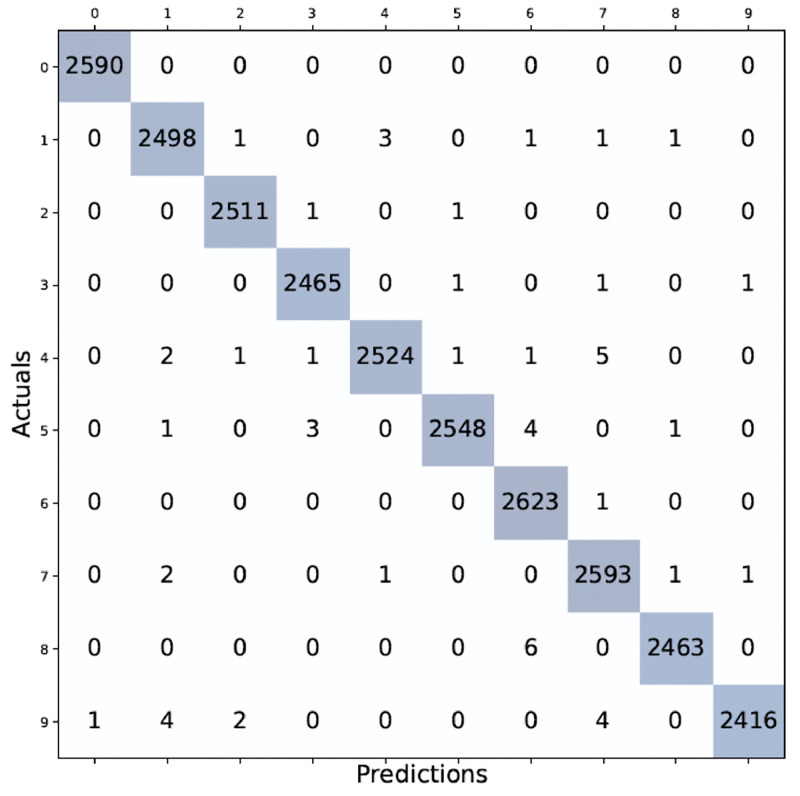
Confusion matrix on DIDA dataset.

To determine the impact of the model on original photos, experiments are also conducted using the DIDA dataset. The average recall, accuracy, precision, and f1-score are 99.81 %. The confusion matrix for the DIDA dataset is depicted in Fig. 7.

**Fig. 8:**
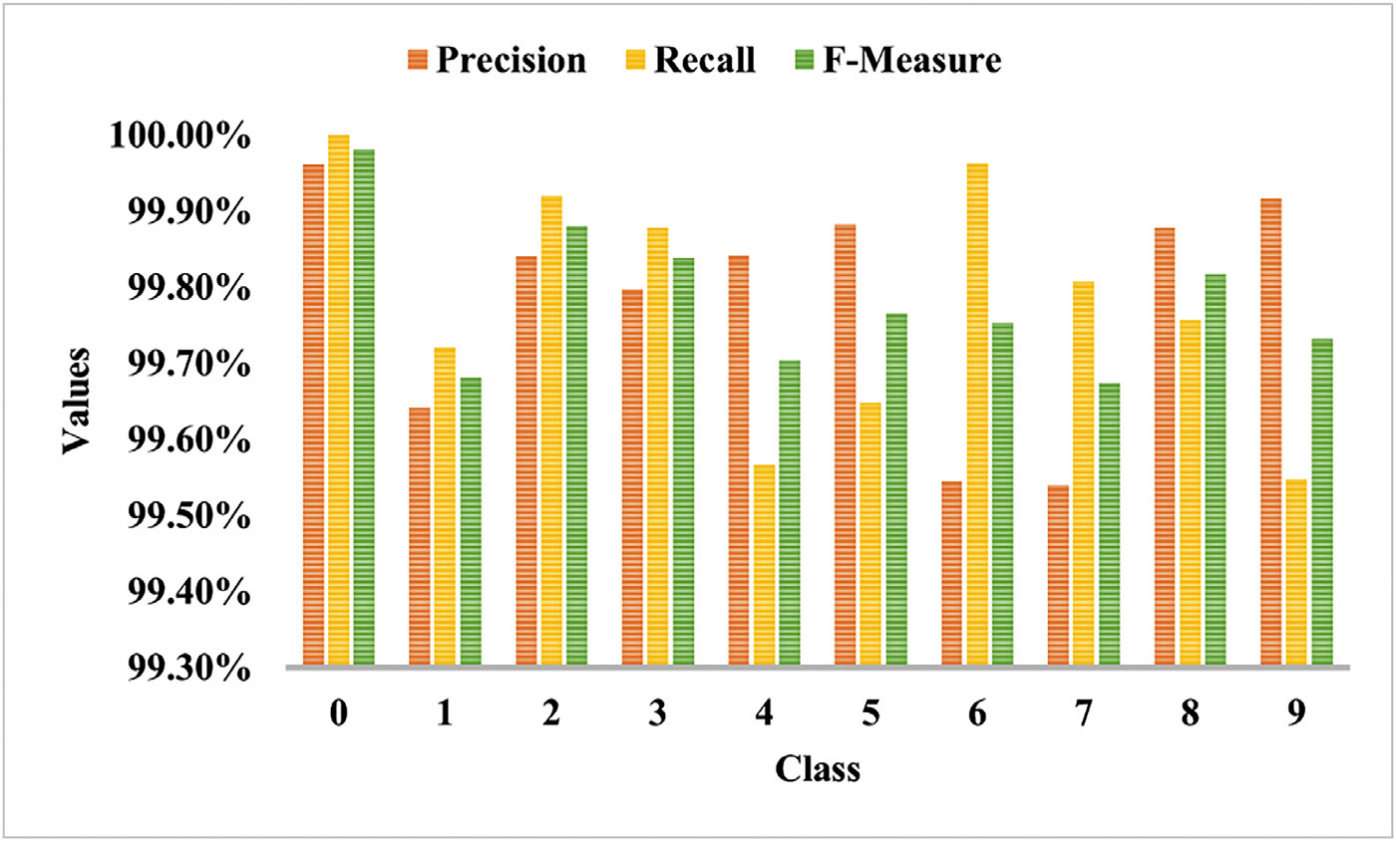
Performance indicators comparison between classes using the DIDA dataset.

Fig. 8 shows the DIDA dataset's classwise precision, recall, and f-measure analysis. As the DIDA dataset is imbalanced, the difference in classwise classification is visible in Fig. 8. The writing styles of 1 and 7 are somewhat similar; hence, they are difficult to classify.

**Fig. 9:**
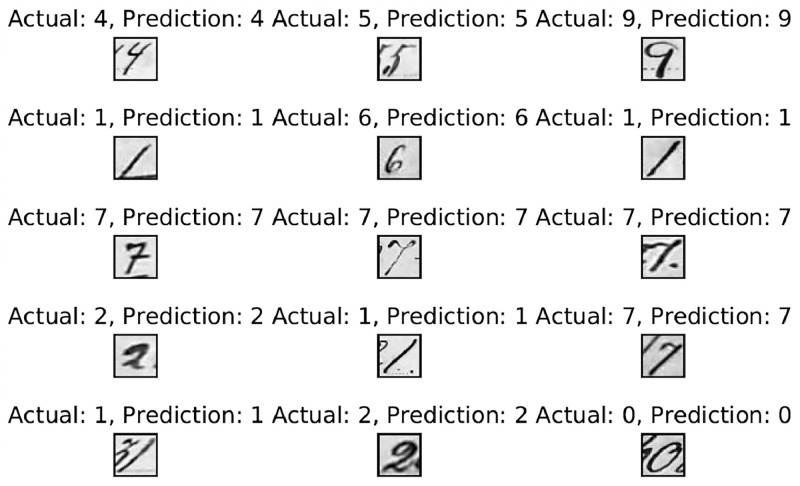
Prediction on handwritten digits of DIDA dataset.

The obtained roc-auc score is 99.99 percent. The outcome of the prediction on a few sample photos is displayed in Fig. 9. As a result, the model can identify numbers in original images.

**Fig. 10:**
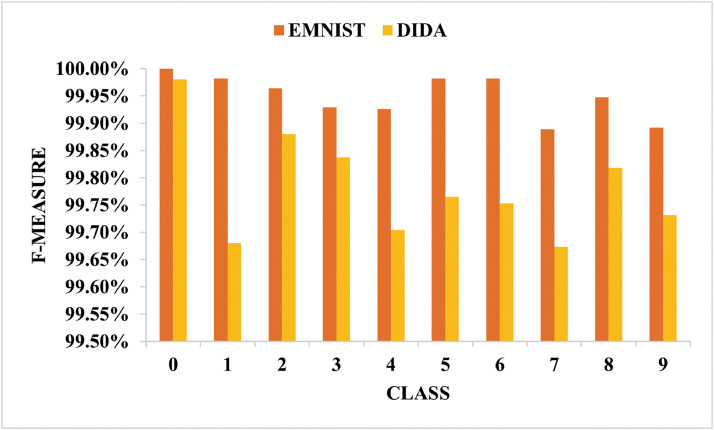
Fold-wise accuracy plot on EMNIST and DIDA datasets.

The fold-wise accuracy on both the datasets, i.e., EMNIST and DIDA, is shown in Fig. 10. Though the DIDA datasets images are uncleaned, the proposed method achieved comparative accuracy like the EMNIST cleaned images dataset. Fig. 10 also depicts that the model is not overfitting.

**Fig. 11:**
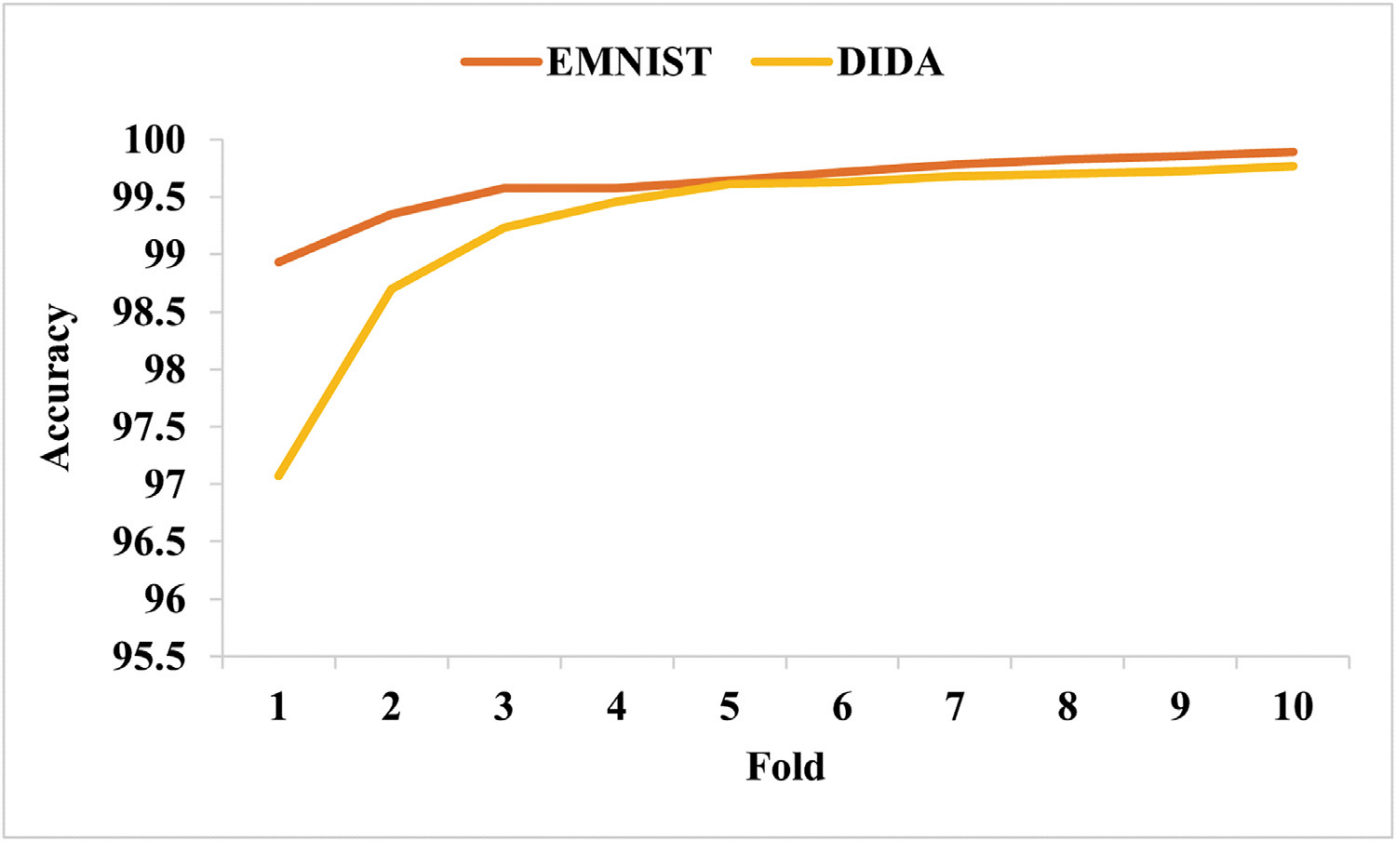
Comparison of the EMNIST and DIDA datasets’ classwise f-measures.

The efficient model must be capable of differentiating among the digits classes. Hence, Fig. 11 compares the classwise f-measure between the EMNIST and DIDA datasets. The Fig. 11 clearly shows that the model is robust and can distinguish digits effectively.

**Fig. 12:**
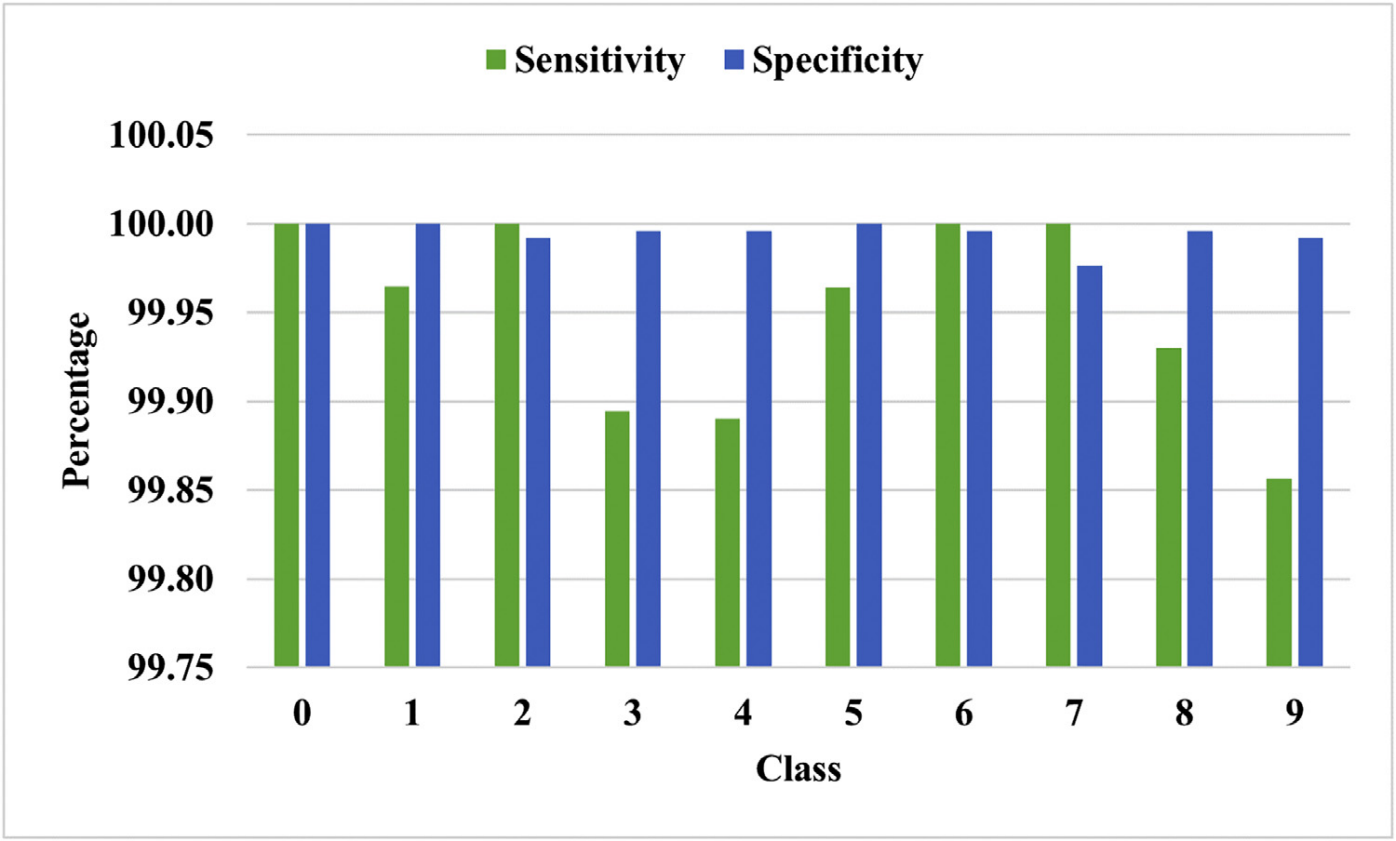
On the EMNIST digit, class-wise sensitivity and specificity.

**Fig. 13:**
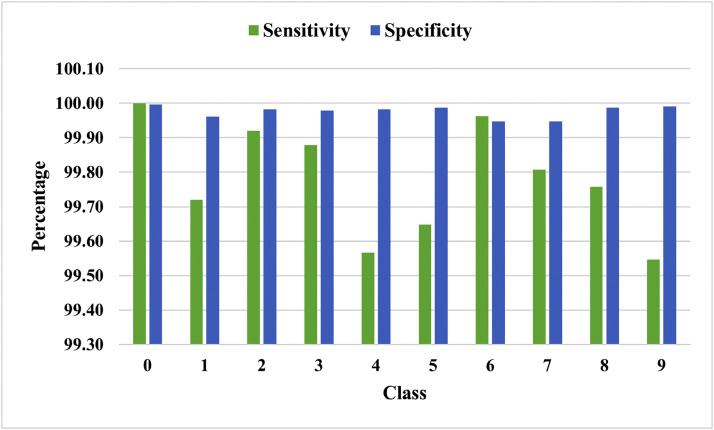
Classwise sensitivity and specificity on DIDA dataset.

The ability of the framework to appropriately measure the positive cases is termed sensitivity, and the power of the algorithm to recognize examples of the harmful category appropriately is measured by specificity. Both dataset's class-wise sensitivity and specificity results are displayed in Fig. 12, Fig. 13. The experiment shows that the framework can accurately predict and categorize positive and negative values.

Table 1 compares the performance of the suggested technique with different architectures on the EMNIST dataset. Comparing the proposed methodology to state-of-the-art methods, the accuracy was greater. Thus, jobs requiring handwriting recognition can be completed using vision transformers. Also, the model results on uncleaned images imply that the attention mechanism is robust to noise and effective in different writing styles. Hence, in any real-time scenario, it can recognize handwritten data such as pin code and date recognition from postal letters and historical documents, respectively.

**Table 1:** Proposed framework accuracy compared to other state-of-the-art methods on EMNIST-digit dataset.

Method	Year	Accuracy (%)
OPIUM [7]	2017	95.90
EDEN [10]	2017	99.30
OptConv+Log+Perc [11]	2020	99.43
CNN [12]	2018	99.46
Parallelized CNN [13]	2017	99.62
Neuro Evolved CNN [14]	2019	99.73
Markov random field CNN [15]	2017	99.75
WaveMixLite-112/16 [16]	2022	99.77
Deep CNN [17]	2018	99.79
TextCaps [18]	2019	99.79
Proposed method	2023	99.89

### Future research directions

**Fig. 14:**
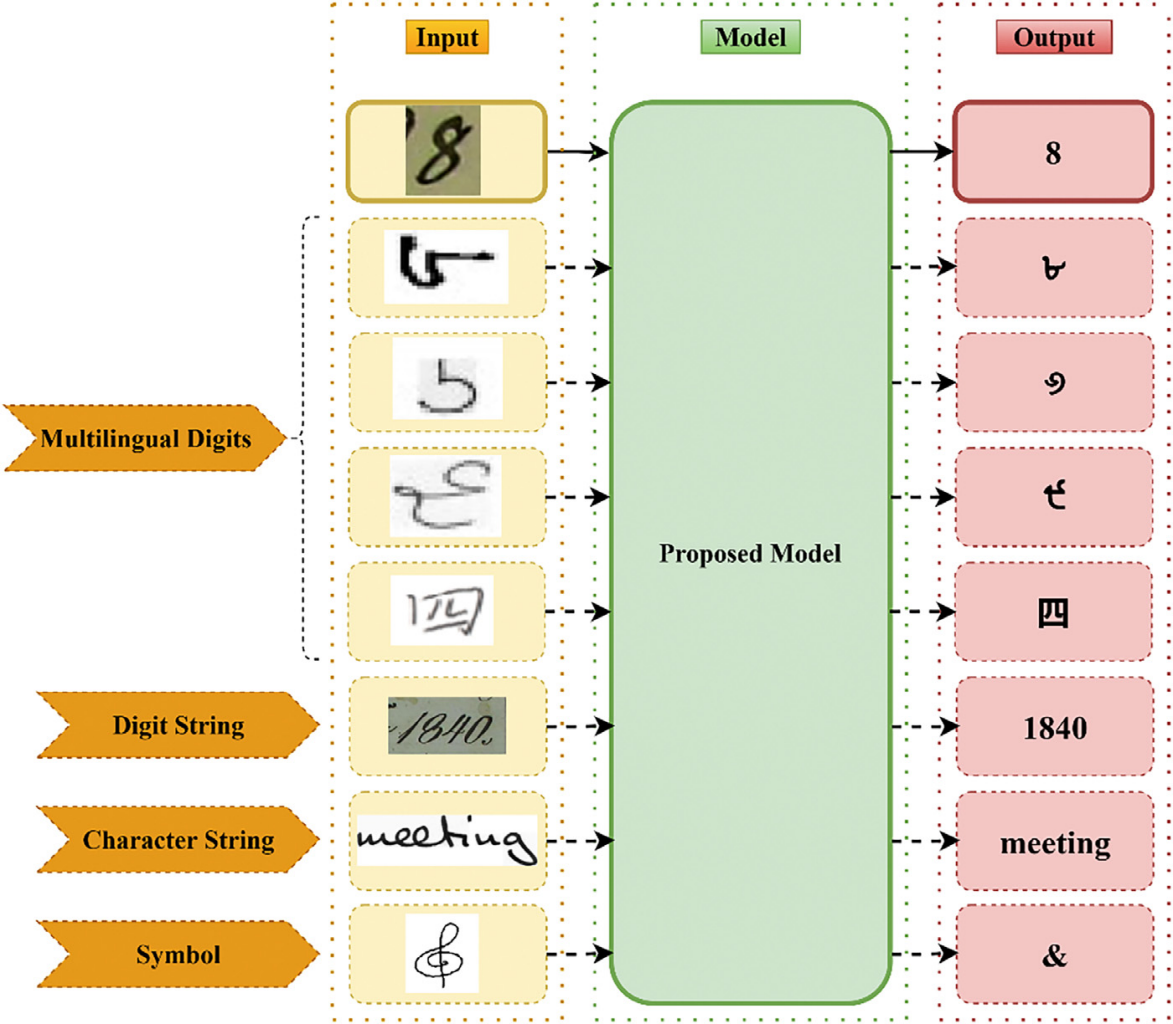
Future research directions of the proposed model.

The proposed model can be generalized for recognizing multilingual handwritten digits and symbols, as shown in Fig. 14. The solid line in Fig. 14 indicates the proposed work and the dashed line indicates future experimentation with the proposed model. Recognition can be done by training the proposed model on multilingual and symbol datasets.

The proposed model can be integrated with decoders to recognize handwritten digit strings and words without character segmentation. Such models can be used in applications like identifying courier addresses, digitizing historical documents, processing bank cheques, etc. The performance of the proposed model can be improved further by using techniques such as hybrid optimizers.

Integrating the proposed method with a graph attention network (GAN) will recognize the handwritten mathematical expressions. Also, the model can be further enhanced to recognize hand-drawn flowcharts, electrical circuit diagrams, molecule symbols, etc. This will be useful, especially in distance education, where students learn online with the help of recent technologies.

## CRediT authorship contribution statement

**Vanita Agrawal:** Conceptualization, Methodology, Data curation, Writing – original draft, Writing – review & editing. **Jayant Jagtap:** Supervision. **Shruti Patil:** Writing – review & editing. **Ketan Kotecha:** Writing – review & editing.

## Declaration of Competing Interest

The authors declare that they have no known competing financial interests or personal relationships that could have appeared to influence the work reported in this paper.

## Data Availability

Data will be made available on request.
